# Supplementary material for: Revealing Key Dimensions Underlying the Recognition of Dynamic Human Actions
Source: Commun Psychol. 2025 Oct 23;3:149. doi: 10.1038/s44271-025-00338-y (PMC12550022; doi:10.1038/s44271-025-00338-y)
Supplement: Supplementary file 2 — Supplementary Information [file 44271_2025_338_MOESM2_ESM.pdf]

Revealing Key Dimensions Underlying the Recognition of Dynamic Human Actions

Supplementary Information

André Bockes[1], Martin N. Hebart[2,3,4] & Angelika Lingnau[1,4]

1. Chair of Cognitive Neuroscience, University of Regensburg, Regensburg, Germany

2. Computational Cognitive Neuroscience and Quantitative Psychiatry, Justus Liebig University, Gießen, Germany

3. Center for Mind, Brain and Behavior, Universities of Marburg, Gießen, and Darmstadt, Germany

4. Vision and Computational Cognition Group, Max Planck Institute for Human Cognitive and Brain Sciences, Leipzig, Germany

Stimuli

Supplementary Table 1 | List of action categories reflected by stimuli.

Overall, 256 action categories were expressed through the stimulus dataset used in this study. Note that from the original 339 action categories, not all were included. Categories that were subdivided by age and gender of the actor (e.g. "adult+female+singing") were combined into one single category ("singing").

#	Action category	#	Action category	#	Action category	#	Action category	#	Action category
1	aiming	53	crafting	105	hitchhiking	157	punting	209	spraying
2	applauding	54	cramming	106	hitting	158	pushing	210	spreading
3	arresting	55	crawling	107	howling	159	racing	211	sprinkling
4	ascending	56	crushing	108	hugging	160	rafting	212	sprinting
5	asking	57	crying	109	hunting	161	raising	213	squatting
6	assembling	58	cuddling	110	inflating	162	reading	214	squinting
7	autographing	59	cutting	111	injecting	163	repairing	215	stacking
8	baking	60	dancing	112	interviewing	164	riding	216	standing
9	balancing	61	descending	113	jogging	165	rinsing	217	steering
10	baptizing	62	digging	114	juggling	166	rising	218	stirring
11	barbecuing	63	dining	115	jumping	167	rocking	219	stitching
12	bathing	64	dipping	116	kicking	168	rolling	220	stomping
13	bending	65	discussing	117	kissing	169	rowing	221	stretching
14	bicycling	66	diving	118	knocking	170	running	222	studying
15	biting	67	draining	119	laughing	171	sailing	223	submerging
16	blowing	68	drawing	120	lecturing	172	saluting	224	surfing
17	boarding	69	dressing	121	licking	173	sanding	225	sweeping
18	boating	70	drilling	122	lifting	174	sawing	226	swimming
19	bouncing	71	drinking	123	loading	175	scratching	227	swinging
20	bowing	72	driving	124	locking	176	screwing	228	taping
21	boxing	73	drying	125	marching	177	serving	229	tapping
22	breaking	74	dusting	126	marrying	178	sewing	230	tattooing
23	brushing	75	eating	127	massaging	179	shaving	231	teaching
24	bubbling	76	emptying	128	measuring	180	shooting	232	telephoning
25	building	77	entering	129	mopping	181	shopping	233	tickling
26	burying	78	exercising	130	opening	182	shouting	234	towing
27	buttoning	79	exiting	131	operating	183	shoveling	235	trimming
28	buying	80	falling	132	packaging	184	shredding	236	tripping
29	calling	81	feeding	133	packing	185	shrugging	237	tuning
30	camping	82	fighting	134	painting	186	signing	238	twisting
31	carrying	83	filling	135	parading	187	singing	239	tying
32	carving	84	filming	136	paying	188	sitting	240	typing
33	catching	85	fishing	137	pedaling	189	skating	241	unloading
34	celebrating	86	flicking	138	peeling	190	sketching	242	unpacking
35	cheering	87	flipping	139	performing	191	skiing	243	vacuuming
36	cheerleading	88	floating	140	photographing	192	slapping	244	waking
37	chewing	89	flying	141	picking	193	sleeping	245	walking
38	chopping	90	folding	142	piloting	194	slicing	246	washing
39	clapping	91	frowning	143	planting	195	sliding	247	watering
40	clawing	92	frying	144	playing+fun	196	slipping	248	waving
41	cleaning	93	fueling	145	playing+music	197	smashing	249	weeding
42	climbing	94	gardening	146	playing+sports	198	smelling	250	wetting
43	clipping	95	giving	147	plugging	199	smiling	251	whistling
44	closing	96	grilling	148	plunging	200	smoking	252	working
45	coaching	97	gripping	149	pointing	201	snapping	253	wrapping
46	combing	98	grooming	150	pouring	202	sniffing	254	wrestling
47	combusting	99	guarding	151	praying	203	snuggling	255	writing
48	competing	100	hammering	152	preaching	204	sowing	256	yawning
49	constructing	101	handcuffing	153	pressing	205	speaking		
50	cooking	102	handwriting	154	protesting	206	spilling		
51	coughing	103	hanging	155	pulling	207	spitting		
52	cracking	104	hiking	156	punching	208	splashing		

Task

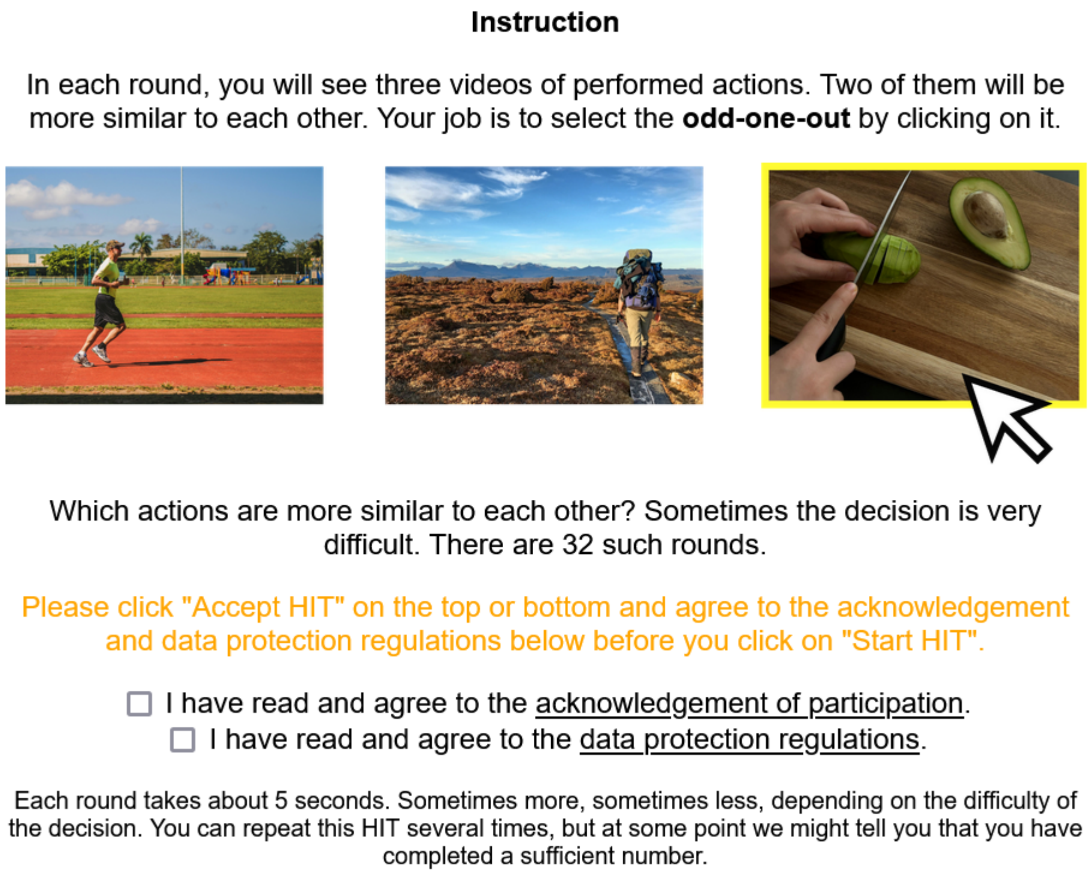

Supplementary Fig. 1 | Instruction for triplet-odd-one-out online task.

The triplet-odd-one out task was carried out on the platform Amazon Mechanical Turk (AMT) in sets of 32 click decisions. Preceding each set of 32, the depicted instruction screen was displayed. As a visual example, the triplet ‘running’/ ’hiking’/ ’cutting’ was shown, together with the general instructions. The triplet and the suggested odd-one-out choice (‘cutting’) was chosen to be both explanatory and comprehensive for the triplet-odd-one-out task. The example was the same for all displayed instruction screens and was possible to also be included among presented triplets during data acquisition. Moreover, before starting the experiment, participants had to consent to their participation and to the data protection regulations via mouse click. The instruction was intentionally kept relatively short and visual to both communicate the task intuitively yet also facilitating multiple participations by participants.

For this figure, the original MiT video frames were replaced by images under a free to use license (Pexels: Caleb Oquendo, Esranur Kalay, Mark Gleeson).

Dimensions

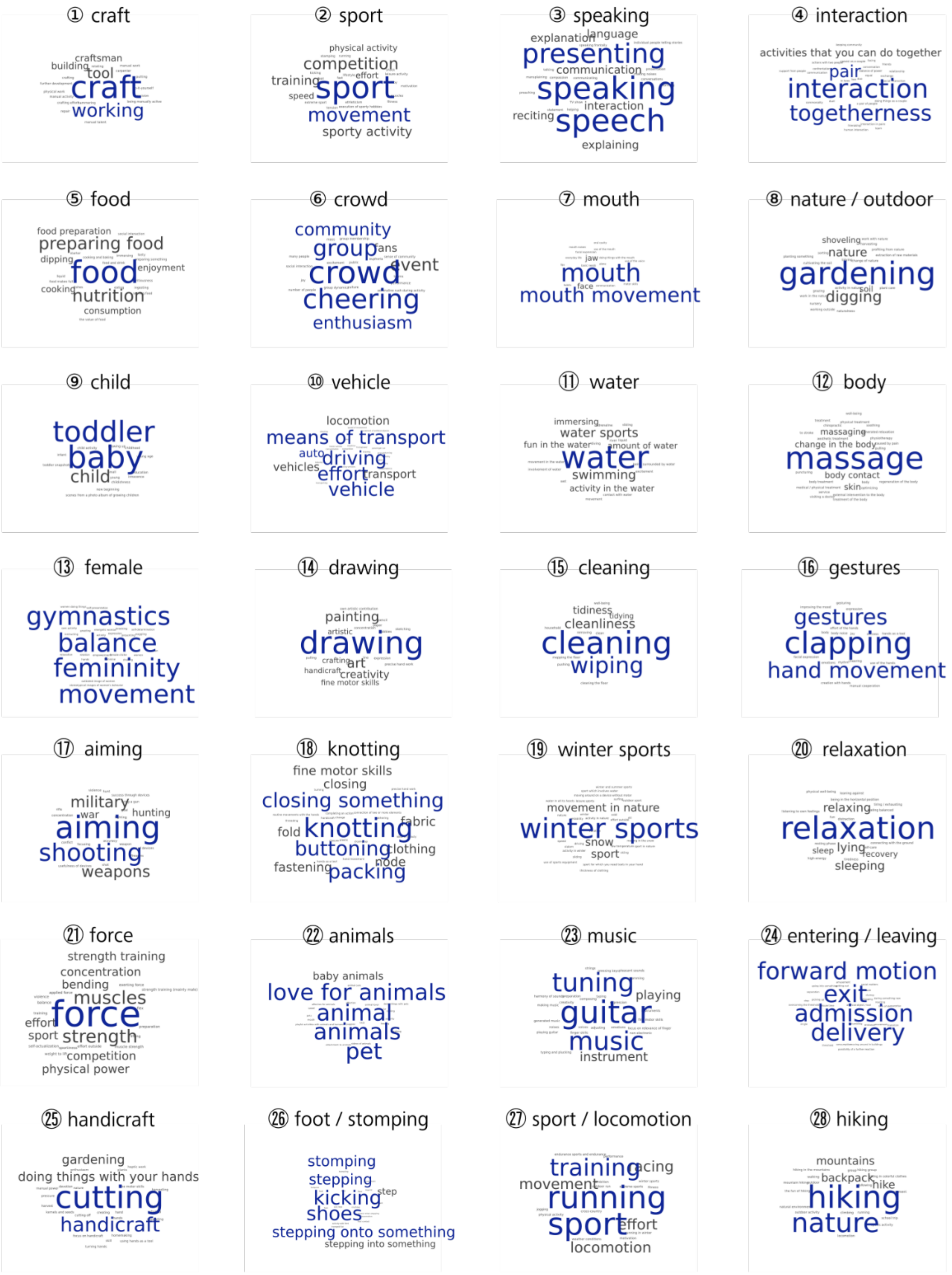

① craft
craftsman
building
tool
craft
working
② sport
physical activity
competition
training
sport
speed
movement
sporty activity
③ speaking
explanation
language
presenting
communication
speaking
interaction
reciting
speech
explaining
④ interaction
activities that you can do together
pair
interaction
togetherness
⑤ food
food preparation
preparing food
dipping
food
enjoyment
cooking
nutrition
consumption
⑥ crowd
community
group
fans
crowd
event
cheering
enthusiasm
⑦ mouth
jaw
mouth
face
mouth movement
⑧ nature / outdoor
shoveling
nature
gardening
soil
digging
⑨ child
toddler
baby
child
⑩ vehicle
locomotion
means of transport
auto
driving
vehicles
effort
transport
vehicle
⑪ water
immersing
water sports
fun in the water
amount of water
water
swimming
activity in the water
⑫ body
massaging
change in the body
massage
body contact
skin
⑬ female
gymnastics
balance
femininity
movement
⑭ drawing
painting
artistic
drawing
crafting
art
handicraft
creativity
fine motor skills
⑮ cleaning
tidiness
tidying
cleanliness
cleaning
wiping
⑯ gestures
gestures
clapping
hand movement
⑰ aiming
military
war
hunting
aiming
shooting
weapons
⑱ knotting
fine motor skills
closing
closing something
fold
knotting
fabric
buttoning
clothing
fastening
node
packing
⑲ winter sports
movement in nature
winter sports
snow
sport
⑳ relaxation
relaxing
relaxation
sleep
lying
recovery
sleeping
㉑ force
strength training
concentration
bending
muscles
effort
force
sport
strength
competition
physical power
㉒ animals
baby animals
love for animals
animal
animals
pet
㉓ music
tuning
playing
guitar
music
instrument
㉔ entering / leaving
forward motion
exit
admission
delivery
㉕ handicraft
gardening
doing things with your hands
cutting
handicraft
㉖ foot / stomping
stomping
stepping
kicking
step
shoes
stepping onto something
stepping into something
㉗ sport / locomotion
training
racing
movement
running
sport
effort
locomotion
㉘ hiking
mountains
backpack
hike
hiking
nature

Supplementary Fig. 2 | Naming word clouds for all 28 action embedding dimensions. Word clouds depicting all 28 action embedding dimensions. These labels were generated by a separate group of 21 laboratory participants after visually examining each dimension. Note that the most frequently named label (indicated by the largest font and blue color) was not always considered to be the most suitable name representing the whole dimension (see also examples in Fig. 3). Font size corresponds to the naming frequency of labels over all participants. The dimensions display various characteristics that are relevant to judge the similarity of actions, including properties related to goal-directed actions, living- and non-living things, environment, substance, and force.

Supplementary Table 2 | List of dimension names.

List of the 28 dimensions sorted in descending order by the sum of their weights among videos, together with the corresponding labels provided by the authors (2nd column) and by a large language model (Chat-GPT-4) that was provided with dimension naming data obtained from a separate group of participants (3rd column; see Methods for details). Note that the ordering places dimensions which are more important for the SPoSE model at the top of the list.

Dimension number	Dimension name (authors)	Dimension description (Chat-GPT-4)
1	craft	manual labor and craftsmanship
2	sport	sports and physical activity
3	speaking	communication and speech
4	interaction	pair interaction and togetherness
5	food	food and cooking
6	crowd	group dynamics and public events
7	mouth	mouth and oral activities
8	nature / outdoor	gardening and nature activities
9	child	babies and early childhood
10	vehicle	automotive and transportation concepts
11	water	water activities and sports
12	body	body care and aesthetics
13	female	empowerment and feminine expression
14	drawing	visual arts and creative expression
15	cleaning	household cleaning and tidying
16	gestures	gesture and expression through hand movements
17	aiming	marksmanship and weaponry
18	knotting	fine motor skills and handicraft techniques
19	winter sports	winter outdoor activities and sports
20	relaxation	rest and relaxation
21	force	physical strength and training
22	animals	animal companionship and care
23	music	music creation and instrumental skills
24	entering / leaving	transition and movement in social contexts
25	handicraft	handicrafts and gardening
26	foot / stomping	foot movement and locomotion
27	sport / locomotion	endurance sports and running
28	hiking	hiking and outdoor exploration

Supplementary Table 3 | Comparison of dimensions revealed in the current study and those obtained in previous studies.

Overview of the dimensions revealed in the current study (second row) and the broad categories they can be assigned to (top row). Dimensions with the highest weights (top 10) are highlighted in bold. The remaining rows show similar dimensions identified in previous studies that focused on actions (rows 3-7) and objects (row 8).

Human actions				Living things		Non-living things		Environment	Substance	Force	Study
Instrumental / goal-directed	Communicative / Social	Locomotion / Sport-related	Expressive / Leisure actions	Whole	Part	Manipulable	Non-manipulable				
**(1) Craft**<br>(15) Cleaning<br>(18) Knotting<br>(25) Handicraft	**(3) Speaking**<br>**(4) Interaction**<br>(16) Gestures	**(2) Sport**<br>(17) Aiming<br>(19) Winter Sports<br>(24) Entering / Leaving<br>(27) Sport / Locomotion<br>(28) Hiking	(14) Drawing<br>(20) Relaxation<br>(23) Music	**(6) Crowd**<br>**(9) Child**<br>(13) Female<br>(22) Animals	**(7) Mouth**<br>(12) Body<br>(26) Foot / Stomping	**(5) Food**	**(10) Vehicle**	**(8) Nature / Outdoor**	(11) Water	(21) Force	Bockes et al.<br>(2024)
Work / Working<br>Chores<br>Cleaning	Talking		Learning<br>Reading<br>Games<br>Celebration<br>Sleeping	People<br>Children		Eating		Nature<br>Outdoors			Dima et al.<br>(2023)
Household-related actions<br>Morning routine<br>Hand-related actions	Communication<br>Interaction<br>Gestures<br>Aggressive actions	Sport-related actions<br>Locomotion	Hobby			Food-related actions					Kabulska et al.<br>(2022)
Creation<br>(Creation / Crime)<br>Spiritualism<br>(Work / Worship)			Spiritualism<br>(Work / Worship)	Animacy<br>(Animate / Mechanical)		Food<br>(Food / Nonfood)					Thornton et al.<br>(2021)
Cleaning-related	Social / Communicative	Locomotion	Leisure-related			Food-related					Tucciarelli et al.<br>(2019)
Unplanned / Planned			Unfriendly / Friendly							Feeble / Formidable	Vinton et al.<br>(2023)
Construction-related / Physical work-related		*Sports-related*		*Animal-related*<br>*Feminine*<br>*Disgusting / Bugs*<br>*Child-related*	*Body part-related*<br>*Arms / Legs / Skin-related*<br>*Head-related / Face-related*	*Food / Eating-related*<br>*Plant-related / Green*<br>*Clothing-related*<br>*Wood-related*<br>*Tool-related*<br>*Paper-related*<br>*Musical Instrument-related*<br>*Container-related*<br>*Medicine-related*<br>*Handicraft-related*	*Furniture-related / Household-related*<br>*Transportation*	*Outdoors-related*<br>*Bathroom-related*<br>*Sky-related*	*Fluid-related / Drink-related*<br>*Water-related*		*Hebart et al.*<br>*(2020)*<br><br>*(for objects)*

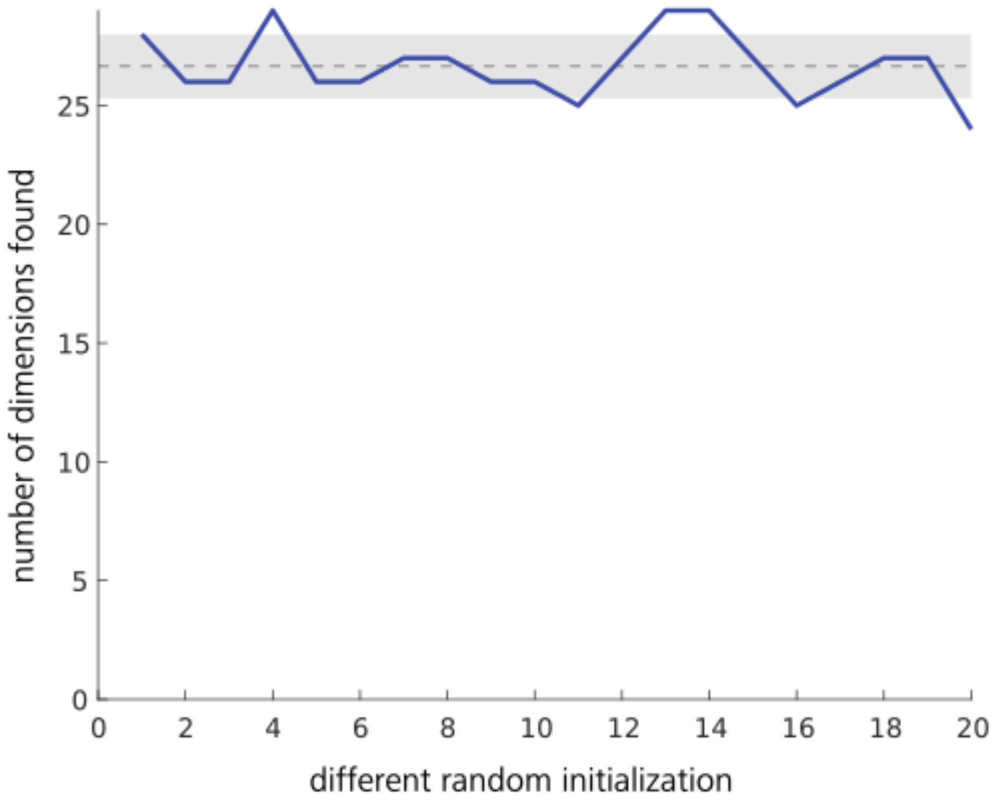

Supplementary Fig. 3 | Stability of embedding dimension number.

At the beginning of the SPoSE modeling procedure a weight matrix from which the final dimension embeddings were generated was initialized randomly. Therefore, potentially slightly different results could have been expected due to the stochastic nature of this process. To check this impact, we additionally ran 20 individual SPoSE models, using our optimal hyperparameter ($\lambda = 0.008$). Each initialization was using a different random seed. Depicted is the number of dimensions against the number of random initializations as a dark blue line. The average number of extracted dimensions was 26.65, as indicated by the dotted line. The gray area depicts the standard deviation. Our final model (random initialization one in this figure) extracted 28 dimensions, displaying approximately one dimension more than the average estimate.

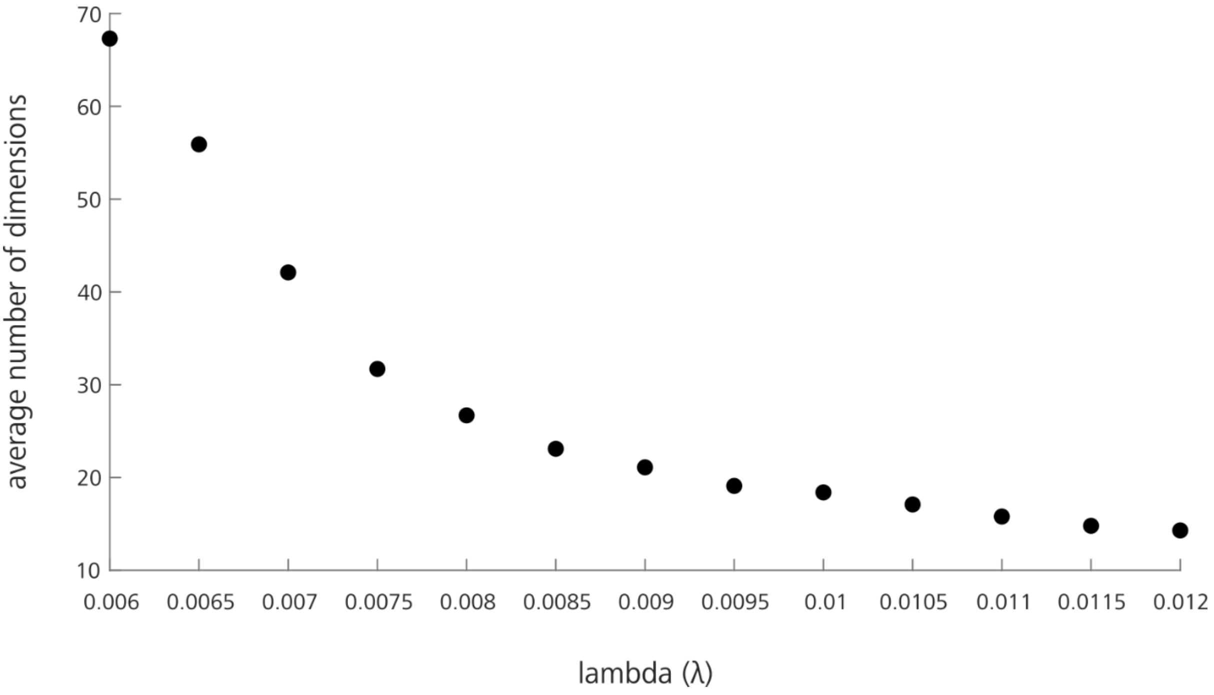

Supplementary Fig. 4 | Influence of hyperparameter lambda on number of dimensions. During the assessment of the optimal hyperparameter lambda (see also Methods, 'Hyperparameter optimization'), 13 different lambda values were chosen, between 0.006 and 0.012 with a step size of 0.005. For each of those 13 values, ten individual optimization procedures (using 10 different random seeds) were run, resulting in 130 modelling processes in total. This scatter plot depicts the average number of obtained embedding dimensions, over the ten optimization procedures for each of the 13 lambda values. As the hyperparameter lambda is controlling the tradeoff between model complexity and sparsity during the SPoSE modelling procedure, the average number of final dimensions changes with respect to chosen value for lambda. For various of those lambda values, corresponding sets of dimensions were visualized through their top stimuli. These dimension visualizations were then compared visually against each other by inspecting the information content of the first few dimensions. The main dimensionality information was found to be contained in each dimension set (with different levels of complexity), suggesting that the same information can be expressed through multiple sets of dimensions with varying numbers of dimensions.

Supplementary Table 4: Comparison of dimensions underlying action recognition (current study) and object recognition (Hebart et al., 2020) for dimensions 1-10. Columns 2 and 4 depict dimension labels, columns 3 and 5 show model weight percentages. Similar or very closely related dimensions that were obtained in both models within the top 10 dimensions are highlighted in bold font.

	**Action recognition** (current study) 28 dimensions		**Object recognition** (Hebart et al., 2020) 49 dimensions	
Dimension number	label	[%]	label	[%]
1	Craft	11.1	Made of metal / artificial / hard	8
2	Sport	10.7	**Food-related / eating-related / kitchen-related**	6.3
3	Speaking	7.6	Animal-related / organic	4.7
4	Interaction	5.2	Clothing-related / fabric / covering	3.4
5	**Food**	4.9	Furniture-related / household-related / artifact	3.3
6	Crowd	4.8	Plant-related / green	3.2
7	**Mouth**	4.7	**Outdoors-related**	2.8
8	**Nature / Outdoor**	4.5	**Transportation / motorized / dynamic**	2.8
9	Child	4.2	Wood-related / brown	2.8
10	**Vehicle**	4.2	**Body part-related**	2.7
