# Supplementary material for: Revealing Key Dimensions Underlying the Recognition of Dynamic Human Actions
Source: Commun Psychol. 2025 Oct 23;3:149. doi: 10.1038/s44271-025-00338-y (PMC12550022; doi:10.1038/s44271-025-00338-y)
Supplement: Supplementary file 3 — Bockes et al nr-reporting-summary [file 44271_2025_338_MOESM3_ESM.pdf]

nature portfolio

Corresponding author(s): Angelika Lingnau

Last updated by author(s): Sep 4, 2025

Reporting Summary

Nature Portfolio wishes to improve the reproducibility of the work that we publish. This form provides structure for consistency and transparency in reporting. For further information on Nature Portfolio policies, see our Editorial Policies and the Editorial Policy Checklist.

Statistics

For all statistical analyses, confirm that the following items are present in the figure legend, table legend, main text, or Methods section.

n/a	Confirmed	
☐	☒	The exact sample size (*n*) for each experimental group/condition, given as a discrete number and unit of measurement
☒	☐	A statement on whether measurements were taken from distinct samples or whether the same sample was measured repeatedly
☒	☐	The statistical test(s) used AND whether they are one- or two-sided<br>*Only common tests should be described solely by name; describe more complex techniques in the Methods section.*
☒	☐	A description of all covariates tested
☒	☐	A description of any assumptions or corrections, such as tests of normality and adjustment for multiple comparisons
☒	☐	A full description of the statistical parameters including central tendency (e.g. means) or other basic estimates (e.g. regression coefficient) AND variation (e.g. standard deviation) or associated estimates of uncertainty (e.g. confidence intervals)
☒	☐	For null hypothesis testing, the test statistic (e.g. *F*, *t*, *r*) with confidence intervals, effect sizes, degrees of freedom and *P* value noted<br>*Give P values as exact values whenever suitable.*
☒	☐	For Bayesian analysis, information on the choice of priors and Markov chain Monte Carlo settings
☒	☐	For hierarchical and complex designs, identification of the appropriate level for tests and full reporting of outcomes
☐	☒	Estimates of effect sizes (e.g. Cohen's *d*, Pearson's *r*), indicating how they were calculated

Our web collection on statistics for biologists contains articles on many of the points above.

Software and code

Policy information about availability of computer code

Data collection	/see Methods, section Data collection<br><br>• Online experiments: Custom HTML, CSS and Javascript code.<br>• Laboratory experiments: Custom Matlab code.
Data analysis	/see Methods, section Data analysis<br><br>Custom Matlab code (Version R2018a).

For manuscripts utilizing custom algorithms or software that are central to the research but not yet described in published literature, software must be made available to editors and reviewers. We strongly encourage code deposition in a community repository (e.g. GitHub). See the Nature Portfolio guidelines for submitting code & software for further information.

Data

Policy information about availability of data

All manuscripts must include a data availability statement. This statement should provide the following information, where applicable:
- Accession codes, unique identifiers, or web links for publicly available datasets
- A description of any restrictions on data availability
- For clinical datasets or third party data, please ensure that the statement adheres to our policy

Data needed to evaluate the main conclusions in the manuscript is available via an OSF repository (https://osf.io/b79eu/).

Research involving human participants, their data, or biological material

Policy information about studies with human participants or human data. See also policy information about sex, gender (identity/presentation), and sexual orientation and race, ethnicity and racism.

Reporting on sex and gender	Gender information was provided by participants.
Reporting on race, ethnicity, or other socially relevant groupings	No data on race, ethnicity, or other socially relevant groupings was collected.
Population characteristics	/see section Methods, Participants, for details<br><br>• Online experiment: 6,036 workers (2,807 female, 3,221 male, 8 other, mean age: 35.8 years) from the online crowdsourcing platform Amazon Mechanical Turk (AMT) with location USA<br>• Laboratory experiments: 135 participants (114 female, 20 male, 1 other, mean age: 23.4 years) recruited from the University of Regensburg
Recruitment	/see section Methods, Data collection, for details<br><br>• Online experiment: Via Amazon Mechanical Turk (AMT): Workers based in the United States and already having successfully completed over 100 other tasks on the platform AMT(acceptance rate: > 95%)<br>• Laboratory experiments: Recruited at the University of Regensburg: individual personal recruitment and using the SONA system of the University of Regensburg (https://regensburg.sona-systems.com/)
Ethics oversight	Ethics committee of the University of Regensburg

Note that full information on the approval of the study protocol must also be provided in the manuscript.

Field-specific reporting

Please select the one below that is the best fit for your research. If you are not sure, read the appropriate sections before making your selection.

☐ Life sciences ☒ Behavioural & social sciences ☐ Ecological, evolutionary & environmental sciences

For a reference copy of the document with all sections, see nature.com/documents/nr-reporting-summary-flat.pdf

Behavioural & social sciences study design

All studies must disclose on these points even when the disclosure is negative.

Study description	/see Methods section for details<br><br>Quantitive experimental research
Research sample	/see section Methods, Participants, for details<br><br>• Online experiment: 6,036 workers (2,807 female, 3,221 male, 8 other, mean age: 35.8 years) from the online crowdsourcing platform Amazon Mechanical Turk (AMT) with location USA<br>• Laboratory experiments: 135 participants (114 female, 20 male, 1 other, mean age: 23.4 years) recruited from the University of Regensburg, spreading over three individual experiments: (1) a total of 94 participants (88 female, 5 male, 1 other, mean age: 21.6 years) took part in the Stimulus validation; (2) 20 participants (12 female, 8 male, mean age: 29.5 years) took part in a Pilot study preceding online data collection, and (3) 21 participants (14 female, 7 male, mean age: 25.5 years) took part in the Dimension labeling and the Dimension rating experiment following the main experiment.
Sampling strategy	/see section Methods, Participants, for details

Sampling strategy	Sample size was not predetermined. It was based on previous studies and availability of resources. The sample size for laboratory experiment (3) was based on Hebart et al. (2020).
Data collection	/see section Data collection for details<br><br>• Online experiment: Data collection in web browser using Amazon Mechanical Turk (AMT) online platform, no contact between participants and researcher<br>• Laboratory experiments: Data collection via PC with external monitor
Timing	• Online experiment (Triplet odd-one-out experiment): Start: Jun 26 2023; Stop: Jul 31 2023<br>• Laboratory experiments: (1) Stimulus validation: Start: Mar 31 2021; Stop: May 25 2021 (2) Pilot study: Start: Dec 15 2022; Stop: Feb 01 2023 (3) Dimension naming and rating: Start: Aug 29 2023; Stop: Sept 11 2023
Data exclusions	/see section Data analysis, Triplet odd-one-out experiment: data filtering, for details<br><br>• Online experiment: Workers were excluded if they exhibited noticeably fast responses in at least three sets of 32 trials (speed cut-off 12.5% or more responses <900 ms and 50% or more responses <1,200 ms) or if they exhibited highly deterministic responses (50% or larger chance of choosing one option) in at least six sets of trials. Following this step, all individual trials displaying reaction times below 900 ms were excluded. This lead to a final dataset consisting of 3,905 individual workers (1,942 female, 1,956 male, 7 other, mean age: 36.9 years).<br>• Laboratory experiments: No participants or data was excluded.
Non-participation	No participants dropped out.
Randomization	Participants were not allocated into groups.

Reporting for specific materials, systems and methods

We require information from authors about some types of materials, experimental systems and methods used in many studies. Here, indicate whether each material, system or method listed is relevant to your study. If you are not sure if a list item applies to your research, read the appropriate section before selecting a response.

Materials & experimental systems

n/a	Involved in the study
☒	☐ Antibodies
☒	☐ Eukaryotic cell lines
☒	☐ Palaeontology and archaeology
☒	☐ Animals and other organisms
☒	☐ Clinical data
☒	☐ Dual use research of concern
☒	☐ Plants

Methods

n/a	Involved in the study
☒	☐ ChIP-seq
☒	☐ Flow cytometry
☒	☐ MRI-based neuroimaging

Plants

Seed stocks	n.a.
Novel plant genotypes	n.a.
Authentication	n.a.
